# Psychometric Properties of Four Common Clinical Tests for Assessing Hamstring Flexibility in Young Adults

**DOI:** 10.3389/fphys.2022.911240

**Published:** 2022-06-15

**Authors:** Hao Liu, Ying Shen, Yuan Xiong, Hongfei Zhou, Yuchen Mao, Qiangqiang Shen, Wenxia Hong, Mingjian Liu, Yanqian Liu, Li Qiu, Zhijie Zhang, Yanbing Jia

**Affiliations:** ^1^ Rehabilitation Department, JORU Rehabilitation Hospital, Yixing, China; ^2^ Rehabilitation Medicine Center, The First Affiliated Hospital of Nanjing Medical University, Nanjing, China; ^3^ Department of Physical Therapy, Guangdong Work Injury Rehabilitation Hospital, Guangzhou, China; ^4^ Neuro-Rehabilitation Center, JORU Rehabilitation Hospital, Yixing, China; ^5^ Luoyang Orthopedic Hospital of Henan Province, Luoyang, China

**Keywords:** hamstring, flexibility, psychometric property, reliability, assessment

## Abstract

**Objective:** To examine the psychometric properties of four common hamstring muscle flexibility tests involving the straight leg raise (SLR), passive knee extension (PKE), sit and reach test (SRT) and toe touch test (TTT) in young adults.

**Methods:** Forty-three young healthy adults (mean age 27.4 years) were recruited for 3 repeated sessions of hamstring flexibility assessments using the 4 tests mentioned above and the subsequent isokinetic examinations. The first two sessions (S1 and S2) were conducted by two different raters randomly on the first day (D1), and the third session (S3) was conducted by the same rater as S1 3 days later (D4). The next day (D5), the isokinetic performances of knee extensors and flexors of the dominant leg were assessed. To evaluate the interrater (S1 *vs*. S2) and test-retest (S1 *vs*. S3) reliability of hamstring flexibility tests, intraclass correlation coefficients (ICCs), standard errors of measurement, and the minimum detectable differences were calculated. Correlation analyses were performed to study the association of each hamstring flexibility test with the isokinetic muscle function of the knee flexors (H) and extensors (Q), including the peak torque (PT), total amount of work (TW) and average power (AP).

**Results:** Excellent interrater and test-retest reliability of hamstring flexibility tests involving the SLR, PKE, SRT and TTT were confirmed with ICCs ranging from 0.923 to 0.986. Fair correlations were found between the 4 hamstring flexibility tests and the H/Q for the PT at angular speeds of 180°/s (Pearson’s r at 0.330–0.449). In addition, the PKE was fairly correlated with the AP of the hamstring (Pearson’s r = 0.320) and the H/Q for the TW (Pearson’s r = 0.345) and AP (Pearson’s r = 0.386) at angular speeds of 180°/s.

**Conclusions:** This study confirmed that the SLR, PKE, SRT and TTT were reliable flexibility tests for hamstring muscles in young healthy adults, and the PKE might be a more valid outcome measure to predict hamstring injury.

## 1 Introduction

Hamstrings are some of the most vulnerable muscles to strain with a high recurrence rate during various sports in athletes and college-age students and can lead to prolonged absences from sports (Chu and Rho, 2016; Wing and Bishop, 2020). In the American National Collegiate Athletic Association, a total of 1,142 hamstring injuries were reported in the 2009–2010 to 2013–2014 academic years that leading to 3.05 per 1,000 athlete exposures, which defined as one athlete participating in one competition or practice in which he/she was exposed to the possibility of athletic injury (Dalton et al., 2015). Furthermore, almost 1/3 of hamstring strains recurred (Chu and Rho, 2016), and approximately 6.3% resulted in loss of more than 3 weeks of playing time (Dalton et al., 2015). The injuries and the resulting prolonged absences have been reported to significantly reduce sports performance and result in significant financial expenses (Dalton et al., 2015; Shepherd et al., 2017). Thus, identification of the risk factors for hamstring injuries is necessary to prevent their occurrence and reduce the negative impacts.

A number of risk factors have been reported to be associated with hamstring injuries in previous studies, including but not limited to fatigue, old age, previous injury, inadequate warm up and hamstring muscle weakness or muscle imbalances of the thigh (Sherry et al., 2011; Shepherd et al., 2017; van Dyk et al., 2018). In addition, several studies also support a deficit in hamstring flexibility as a risk factor for hamstring injury (Opar et al., 2012; van Dyk et al., 2018). A recent study involving 438 football players who sustained 78 hamstring injuries demonstrated hamstring flexibility measured using passive knee extension range of motion was independently associated with the injury risk (hazard ratio 0.97, 95% confidence interval 0.95–0.99) (van Dyk et al., 2018). Using multiple logistic regression analysis, a study on 36 English Premier League soccer players showed a significant relationship between the risk of hamstring injuries and hamstring flexibility measured with straight leg raise, and the results demonstrated odds for sustaining an injury increased × 1.29 for each 1° decrease in range of hip flexion (Henderson et al., 2010). On the other hand, tightness or shortness of the hamstring could also affect the posture and gait pattern and lead to plantar fasciitis (Bolívar et al., 2013), patellofemoral pain syndrome (White et al., 2009), and lower back pain (Mistry et al., 2014). Hence, it is important to assess the hamstring flexibility to determine the propensity for hamstring injury and other clinical problems.

The choice of a muscle flexibility test must be based on its reliability and functionality (Mayorga-Vega et al., 2014). In clinical assessments, several tests for measuring hamstring flexibility in terms of the straight leg raise (SLR), passive knee extension (PKE), sit and reach test (SRT) and toe touch test (TTT) are commonly used. Although each of these tests has been demonstrated to be a reliable method with the intraclass correlation coefficients (ICCs) above 0.85 for measuring hamstring flexibility in repeated sessions or *via* varying raters (Gnat et al., 2010; Ayala et al., 2012; Hansberger et al., 2019), no study has compared the reproducibility of all four tests in one homogeneous sample. Thus, which test is the most reliable method for hamstring flexibility measurement in college-age students is unknown. Based on the fact that the reliability scores of a measure are population-specific (Kottner et al.,. 2011), the corresponding values for determining the true change of hamstring flexibility using these four tests performed by different raters or in repeated measurements are also not well known due to inadequate research in college-age students.

On the other hand, thigh muscle function or strength asymmetry has been more confident to be considered as the modifiable risk factor for hamstring injuries (Opar et al., 2012; Afonso et al., 2021). A previous meta analysis involved 195 participants demonstrated quadriceps peak torque was consistently associated with the risk of hamstrings injuries with the standardized mean differences at 0.43 and 95% confidence interval at 0.05 to 0.81 (Freckleton and Pizzari, 2013). Yeung and colleagues (2009) identified that the risk of hamstring injuries increased with a decrease in ratios of knee flexor to knee extensor strength known as hamstrings to quadriceps ratios (H/Q). If the ratio is less than 0.6, the sprinters have a 17 times increased risk of hamstrings injury. However, the precise equipments for muscle function assessment such as isokinetic system are expensive and inconvenient to carry out in clinical practice. In contrast, the hamstring flexibility tests are simple to administer, requires minimal skills training and allows an evaluation in a short of time (Ayala et al., 2012; Mayorga-Vega et al., 2014). In this sense, exploring the relationship between each of hamstring flexibility tests and thigh muscle function which was used as a criterion might indicate the most valuable test for identifying hamstring shortness and predicting the potential injury clinically feasible.

The purpose of this study was therefore to examine the psychometric properties of four hamstring flexibility tests and their relationships with isokinetic knee flexion and extension strength in young adults. Understanding the results of the present study would allow a better determination of hamstring shortness and treatment design to prevent hamstring injuries in clinical practice.

## 2 Materials and Methods

This study was approved by the Research Ethics Committee of the JORU Rehabilitation Hospital (No. 20190706B02).

### 2.1 Participants

Forty-three young healthy volunteers (22 were males) were recruited for the study. All subjects were physiotherapy interns (college student in clinical placement) who had the habit of sports at least once a week with uneventful past and present medical conditions. Female participants were not in the ovulation phase (days 10–14) of their menstrual cycle during the study period (Cejudo et al., 2015). Subjects were excluded if they had a history of musculoskeletal or neurological pathology affecting the dominant leg. The dominant leg was defined as the preferred kicking leg.

### 2.2 Experimental Design and Procedures

This study adopted a repeated measures design. All subjects were verbally informed about the procedures of the study before signing written informed consent to participate in the research. In addition to daily activities, the subjects were asked to refrain from sports exercise for at least 7 days before the first test until they completed the study.

In the first day (D1), subjects participated in a 12 min warm-up period followed by two sessions (S1 and S2) of hamstring flexibility assessment of the dominant leg conducted by rater A and rater B randomly 60 min apart. Three days later (D4), the subjects returned for another session (S3) of hamstring flexibility assessment of the dominant leg, which was conducted by the same rater as S1 after the warm-up period. During the warm-up period, subjects performed 5 min of jogging and 7 min of static stretching including 7 different exercises in which they were held at the gentle stretch posture for 30 s and repeated each exercise twice. This warm-up protocol was adopted in the reliability study to reduce the variability and standard error of measurements by avoiding the potential learning effects of hamstring flexibility along the consecutive testing trials and sessions, as well as minimizing the effect of different muscle temperatures on lower extremity muscle flexibility (Cejudo et al., 2015). The next day (D5), the isokinetic muscle function of the knee extensors and flexors of the dominant leg were assessed in all participants by another rater C. All procedures were conducted at the Neuro-rehabilitation Center of JORU Rehabilitation Hospital between 15:00 and 17:00 p.m. on the testing day at room temperature. The three raters were practicing physical therapists and attended a half-day workshop on the standard procedure of hamstring flexibility tests and muscle function evaluation. All raters were blinded to the purpose of the research and to the assessment results from other raters.

### 2.3 Measures

#### 2.3.1 Hamstring Flexibility

Participants were examined wearing sports clothes and without shoes. During the measurement session, the hamstring flexibility of the dominant leg was assessed using 4 clinical tests including the SLR (Ayala et al., 2011), PKE (Gnat et al., 2010), SRT (Ayala et al., 2011) and TTT (Ayala et al., 2011) in a random order. Three trials for each of the tests were performed by the rater in every session, and the mean score was used for reliability analysis. The subjects were allowed to rest for 1 min between trials and 3 min between tests.

**FIGURE 1:**
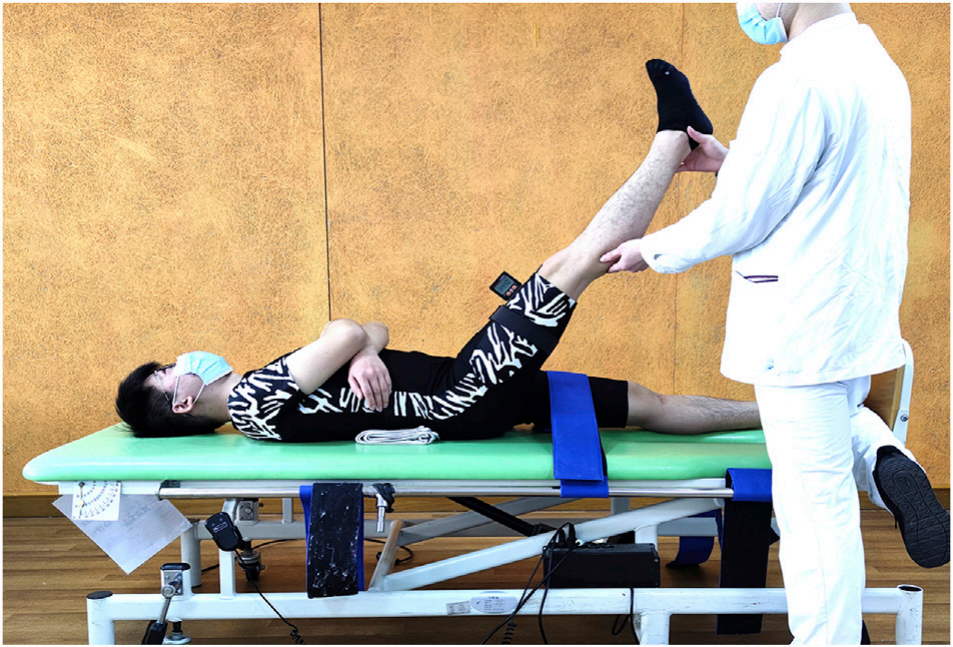
Passive straight leg raise test.

SLR: The subject was lying in the supine position with the lumbar curve supported by a folded towel while the thigh of the nondominant side was restricted with a belt to avoid pelvic tilt and hip flexion. A digital inclinometer (SIWI, Shanghai, China) was placed over the head of the patella in the anterior thigh of the dominant leg. The straight dominant leg was lifted passively by the rater into hip flexion until firm resistance was felt or the subjects stated that they felt the maximum tightness of the posterior of their thigh but without pain. During the procedure, the ankle joint was fixed in a relaxed position. The angle displaced on the screen of the inclinometer was recorded as the hamstring flexibility measure. (Figure 1).

**FIGURE 2:**
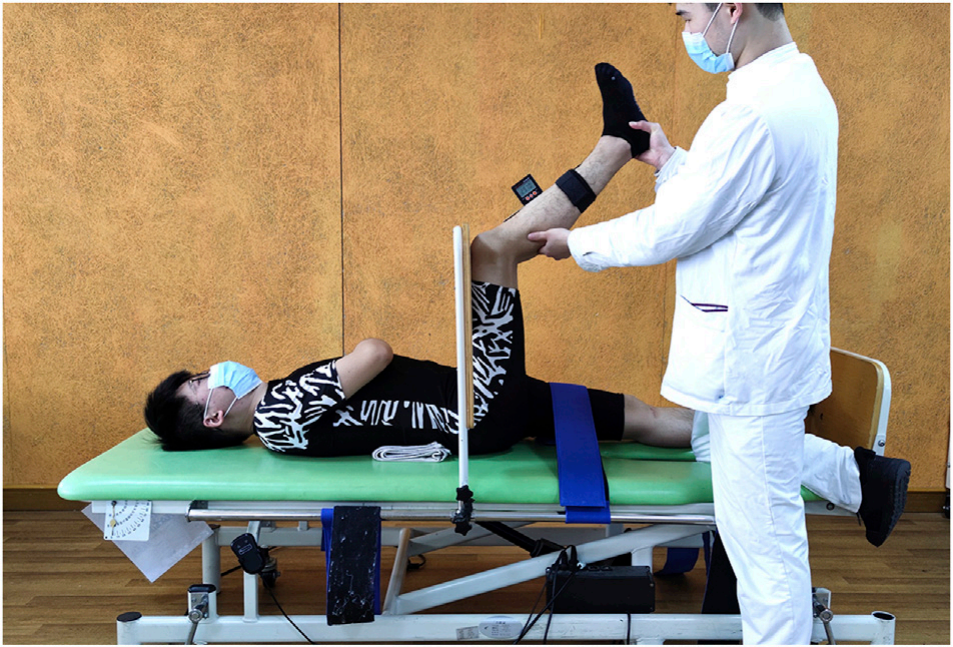
Passive knee extension test.

PKE: The subject was lying in the same way as in the SLR, and the nondominant leg was also restricted. The digital inclinometer was placed over the middle shin on the anterior of the dominant leg. Initially, the dominant leg was passively placed at the hip and knee at 90–90 degree, and the thigh was blocked by a vertical baffle. Then, the rater extended the leg until firm resistance was felt or the subjects stated that they felt the maximum tightness of the posterior of their thigh but without pain. The value of the inclinometer plus 90° was recorded as the total angle of the knee to determine the hamstring flexibility. (Figure 2).

**FIGURE 3:**
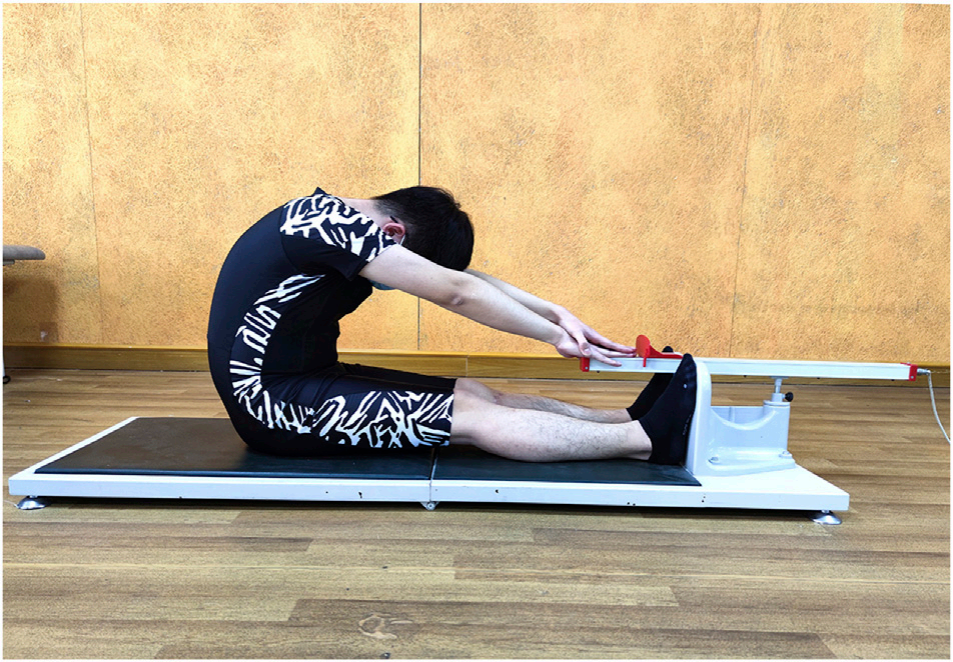
Sit and reach test.

SRT: The subject sat on the floor with their legs straight together and feet placed against the edge of a box, which was defined as the “0” of the nonius scale. Then, the subject was asked to place one hand over the other with their palms down and reach forward to push the nonius as far as possible for approximately 6 s using their middle fingertips. During the test, the subjects were reminded to keep their knee in full extension by the raters. The flexibility of the hamstring was recorded as the value of the distance with the middle fingertips far from the “0” in “+” (away from the trunk) or “-” (close to the trunk) centimeters. (Figure 3).

**FIGURE 4:**
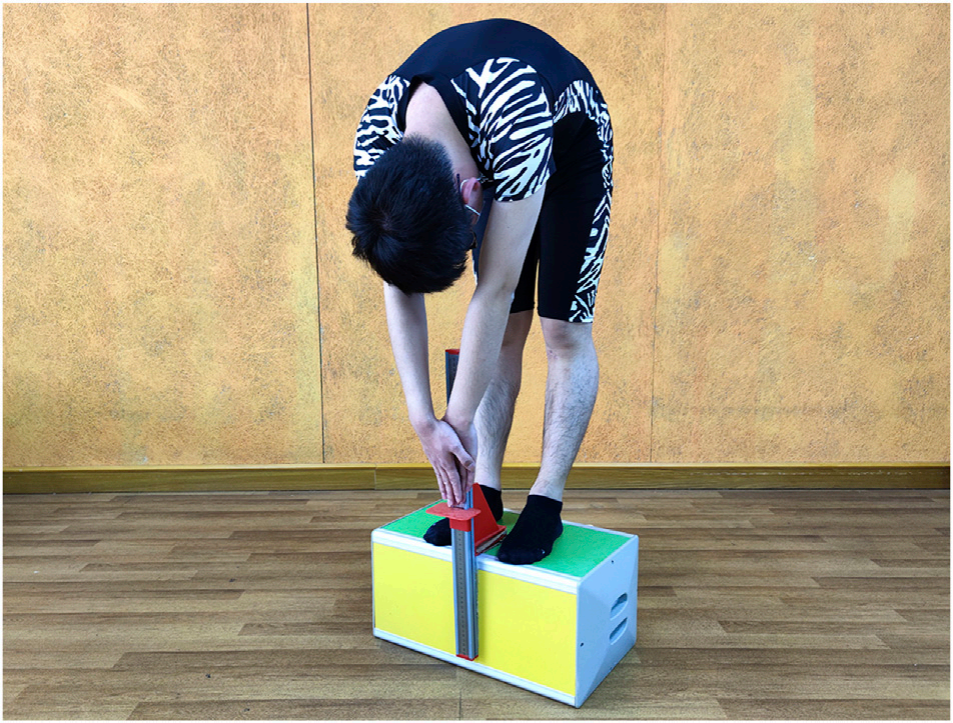
Toe touch test.

TTT: The subject stood on the box with their feet hip-width apart and toes at the edge, which was defined as the “0” of the nonius scale. Then, the subject was asked to place one hand over the other with their palms inward and bend forward to push the nonius as far as possible for approximately 6 s using their middle fingertips. The oral reminder for knee extension was given like SRT. The flexibility of the hamstring was recorded as the value of the distance with the middle fingertips far from the “0” in “+” (away from the trunk) or “-” (close to the trunk) centimeters. (Figure 4).

#### 2.3.2 Muscle Function

The isokinetic muscle strength of the knee extensors and flexors of the dominant leg were evaluated using a Biodex System 4 PRO (Biodex Medical Systems, USA). During the assessment, the subjects were seated on the dynamometer with their chest, waist and thigh stabilized in a coxofemoral flexion of 100° to minimize body movement and avoid compensation. The subjects were instructed to fold their arms across their chest and were not allowed to hold on the chair during the assessment. Then, the seat was adjusted so that the axis of the knee joint of the dominant leg was aligned with the mechanical axis of the test system. After the measurement and correction of the gravitational factor of the dynamometer’s lever arm and lower leg, the ROM of the test knee joint was fixed at a 90° flexion from full extension. After a specific warm-up with 10 consecutive submaximal concentric contractions to become familiar with the movement, the isokinetic performance of knee flexors and extensors was evaluated using concentric/concentric exertions at angular speeds of 60°/s in five maximal repetitions and 180°/s in ten maximal repetitions with 5-min intervals. Parameters including peak torque (PT), total amount of work (TW) and average power (AP) were extracted for flexors (H) and extensors (Q) and the ratio of H/Q in data analysis.

### 2.4 Data Analysis

SPSS statistical software version 23 was used for the data analysis. Means and standard deviations were used to describe the demographic characteristics of subjects and each session of 4 hamstring tests. The interrater and test-retest reproducibility of each test were evaluated for the pairs of S1 *vs*. S2 and S1 *vs*. S3, respectively, which was achieved by calculating the intraclass correlation coefficients (ICCs) using a two-way mixed model (Model 3) and the 95% confidence intervals with a significance level of 0.05. Reliability was defined as poor if the ICC <0.5, moderate if the ICC ≥0.5, good if the ICC ≥0.75 and excellent if the ICC ≥0.9 (Portney and Watkins, 2009).

For the response stability of each flexibility test, the standard error of measurement (SEM) was calculated using the following function:

$${\text{SEM }=\text{ S}}_{x}\surd \left(1-r_{\mathrm{xx}}\right)$$

where S_x_ is the pooled standard deviation and r_xx_ is the reliability coefficient. A smaller SEM indicates less variation of the measurement. According to the SEM, the minimal detectable difference at a 95% confidence interval (MDD_95_), which reflected the minimal change value that can be considered the true change in hamstring flexibility, was calculated as:

$${\text{MDD}}_{95}=1\text{.}96\text{∗SEM∗}\surd 2$$

The relationship between the mean values of a total of 3 sessions of 4 hamstring flexibility tests and muscle function evaluated with the isokinetic system was analyzed using Pearson’s correlation coefficient. The correlation was defined as little or none if |r| < 0.25, fair if 0.25 ≦ |r| < 0.5, moderate to good if 0.5 ≦ |r| < 0.75 and good to excellent if |r| ≥ 0.75 (Portney and Watkins, 2009). The values of r below and above 0 indicate positive and negative correlations, respectively. The significance level was set at 0.05.

## 3 Results

All subjects completed the first day assessment conducted by raters A and B. Subsequently, three subjects and two subjects withdrew from the study after D1 and D4 flexibility assessments, respectively. Thus, the data of 43 subjects were used to calculate the interrater reliability, and the data of 40 subjects were used to determine the test-retest reliability. Only 38 subjects completed the isokinetic test for relationship analysis. The demographic characteristics of all subjects (*n* = 43) are presented in Table 1.

**TABLE 1:** Demographic characteristics of all subjects.

	Healthy Subjects (*n* = 43)
Gender, male:female	22:21
Age (years), mean ± SD	22.35 ± 2.80
Height (cm)	167.95 ± 9.88
Weight (kg)	62.44 ± 12.41
Dominant leg, right:left	41:2

The reliability statistics for each hamstring flexibility test in 3 sessions are presented in Table 2. Excellent interrater (ICC ranged from 0.929 to 0.979) and test-retest (ICC ranged from 0.923 to 0.974) reliability were found for all hamstring flexibility tests. The SEM and MDD_95_ ranged from 3.29° to 3.97° and from 9.12° to 11.00° for the SLR and PKE, respectively; furthermore, they ranged from 1.23 to 1.75 cm and from 3.42 to 4.84 cm for the SRT and TTT, respectively.

**TABLE 2:** Measurement reliability of four common clinical tests for assessing hamstring flexibility in young adults.

Outcomes	Mean ± SD	Mean ± SD	ICC	P	95% CI	SEM	95% CI SEM	MDD_95_
Interrater reliability (*n* = 43): S1 *vs*. S2								
SLR (°)	72.65 ± 15.23	71.05 ± 14.55	0.929	0.000	0.870–0.962	3.97	64.07–79.63	11.00
PKE (°)	148.83 ± 13.42	147.57 ± 11.18	0.929	0.000	0.870–0.962	3.29	141.75–154.65	9.12
SRT (cm)	4.67 ± 10.06	4.88 ± 10.80	0.986	0.000	0.973–0.992	1.23	2.36–7.20	3.42
TTT (cm)	2.27 ± 9.41	3.19 ± 9.65	0.979	0.000	0.959–0.989	1.38	0.02–5.44	3.83
Test-retest reliability (*n* = 40): S1 *vs*. S3								
SLR (°)	73.27 ± 15.53	70.67 ± 15.25	0.944	0.000	0.886–0.971	3.64	64.83–79.10	10.09
PKE (°)	149.30 ± 13.44	147.96 ± 11.67	0.923	0.000	0.856–0.959	3.49	141.78–155.47	9.68
SRT (cm)	4.88 ± 9.86	5.62 ± 10.28	0.974	0.000	0.951–0.986	1.62	2.07–8.44	4.50
TTT (cm)	2.47 ± 9.24	4.38 ± 9.16	0.964	0.000	0.885–0.985	1.75	0.00–6.85	4.84

Abbreviations: ICC, intraclass correlation coefficient; CI, confidence interval; SEM, standard error of measurement; MDD95, minimum detectable difference based on a 95% confidence interval; SLR, straight leg raise test; PKE, passive knee extension; SRT, sit and reach test; and TTT, toe touch test; S1, Session 1; S2, Session 2; S3, Session 3.

The correlation of hamstring flexibility and muscle function is presented in Table 3. A fair correlation was found between the hamstring flexibility tests and H/Q for PT at angular speeds of 180°/s (r = 0.353, *p* = 0.030 for SLR; r = 0.449, *p* = 0.005 for PKE; r = 0.372, *p* = 0.021 for SRT; and r = 0.330, *p* = 0.043 for TTT). In addition, PKE was fairly correlated with the AP of the hamstring (r = 0.320, *p* = 0.050) and H/Q for TW (r = 0.345, *p* = 0.034) and AP (r = 0.386, *p* = 0.017) at angular speeds of 180°/s. The other relationships between hamstring flexibility and parameters of thigh muscle isokinetic performance were not significant.

**TABLE 3:** Relationship between hamstring flexibility measured with 4 common tests and the isokinetic function of thigh muscles.

Isokinetic Muscle Function (mean ± SD) (*n* = 38)			SLR (°) 71.53 ± 14.98		PKE (°) 148.43 ± 11.53		SRT (cm) 4.88 ± 10.32		TTT (cm) 3.27 ± 9.28	
			*r*	*p*	*r*	*p*	*r*	*p*	*r*	*p*
H (60°/s)	PT (N·m)	59.17 ± 27.35	−0.115	0.490	−0.106	0.528	0.117	0.485	0.101	0.544
	TW (J)	299.38 ± 261.48	−0.065	0.700	−0.009	0.956	0.153	0.358	0.145	0.385
	AP (W)	35.99 ± 22.63	0.020	0.904	0.112	0.505	0.225	0.175	0.222	0.181
H (180°/s)	PT (N·m)	38.61 ± 19.12	0.065	0.696	0.169	0.309	0.180	0.279	0.180	0.280
	TW (J)	597.58 ± 466.42	0.087	0.602	0.229	0.166	0.196	0.238	0.264	0.109
	AP (W)	45.75 ± 37.60	0.154	0.356	0.320	0.050*	0.264	0.109	0.277	0.092
Q (60°/s)	PT (N·m)	122.19 ± 44.27	−0.46	0.782	−0.010	0.951	0.054	0.746	0.053	0.752
	TW (J)	577.60 ± 371.11	−0.131	0.432	−0.078	0.643	0.046	0.786	0.049	0.768
	AP (W)	75.82 ± 36.00	−0.053	0.753	0.000	0.998	0.091	0.586	0.089	0.597
Q (180°/s)	PT (N·m)	84.01 ± 36.81	−0.129	0.441	−0.090	0.590	−0.022	0.895	−0.004	0.979
	TW (J)	1,399.53 ± 679.79	−0.030	0.860	−0.105	0.532	−0.029	0.862	0.019	0.912
	AP (W)	108.28 ± 55.60	0.046	0.782	−0.006	0.969	0.032	0.847	0.048	0.774
H/Q (60°/s)	PT (N·m)	0.48 ± 0.12	−0.156	0.350	−0.220	0.185	−0.020	0.906	−0.028	0.866
	TW (J)	0.59 ± 0.77	0.301	0.066	0.250	0.130	0.288	0.079	0.303	0.065
	AP (W)	0.45 ± 0.13	0.155	0.352	0.152	0.363	0.236	0.154	0.233	0.158
H/Q (180°/s)	PT (N·m)	0.46 ± 0.11	0.353	0.030*	0.449	0.005**	0.372	0.021*	0.330	0.043*
	TW (J)	0.41 ± 0.25	0.142	0.394	0.345	0.034*	0.247	0.135	0.292	0.076
	AP (W)	0.39 ± 0.26	0.149	0.373	0.386	0.017*	0.271	0.100	0.285	0.083

Abbreviations: SLR, straight leg raise test; PKE, passive knee extension; SRT, sit and reach test; TTT, toe touch test; H, hamstring; Q, quadriceps; H/Q, the ratio of hamstring to quadriceps; PT, peak torque; TW, total amount of work; and AP, average power.

* Denotes the correlation is significant and *p* < 0.05, and ** denotes the correlation is significant and *p* < 0.01.

## 4 Discussion

The purpose of this study was to examine the psychometric properties of four common hamstring muscle flexibility measures in terms of the SLR, PKE, SRT and TTT in normal young adults of college age and their correlation with the isokinetic performance of the thigh muscles. The results showed that all 4 common hamstring flexibility tests demonstrated excellent repeatability in young healthy subjects. The flexibility of the hamstring, especially that measured by the PKE test, was fairly correlated with the strength balance of the hamstring and quadriceps, which is considered a factor for hamstring injury. To the best of our knowledge, such relationship between hamstring flexibility tests and thigh muscle imbalance has not been reported, although the correlation found in present study is modest. This new finding may support the PKE test to some extent as the most valuable hamstring flexibility test to indicate the risk of hamstring injury for college-age students.

The ICC was considered to reflect the correlation and agreement of repeated measures (Portney and Watkins, 2009). It was therefore regarded as the most accurate statistical index for reliability. The current study found high ICCs above 0.9 for the SLR, PKE, SRT and TTT conducted by different raters and the same rater in two different sessions, which might indicate excellent interrater and test-retest reliability for each of these four hamstring flexibility measures. This result partially agreed with those of previous studies. With a large sample of 243 young adults, Ayala et al. (2012) showed good to excellent test-retest reliability for hamstring flexibility tests with ICCs of 0.85, 0.92 and 0.89 for the SLR, SRT and TTT, respectively. (Carregaro et al., 2007). also demonstrated the excellent interrater (ICC = 0.96) and test-retest (ICC = 0.94) reliability of the SLR for 35 healthy males. In another study of the test-retest design, Cejudo et al. (2015) achieved an ICC of 0.87–0.94 for the SLR on 60 futsal and 30 handball players. Regarding the reliability of the PKE test, Gnat et al. (2010) demonstrated ICCs of 0.93 for both the interrater and test-retest reliability for 14 healthy subjects. The combined results of the current study and these previous studies show that all 4 common clinical tests for assessing hamstring flexibility in young adults are reliable.

SEM was considered to reflect the stability of the response for outcomes (Portney and Watkins, 2009). Thus, a larger SEM represents a larger variation of the test measured in the current study. MDD_95_ is the minimum value of the true change for certain outcomes and could therefore be applied to evaluate the effects of a clinical intervention or changes in the physical status of specific people. In this study, the SEM and MDD_95_ were calculated according to the ICCs in terms of both interrater and test-retest reliability for these 4 hamstring flexibility tests. With the exception of reliability data, the present results also provide an objective indicator for the change in the hamstring flexibility in young adults. For example, with the MDD_95_ for the PKE being 9.12° and 9.68° for the interrater and test-retest measurements respectively, a change in the PKE test greater than these angles would have a 95% probability of being a real change in the hamstring flexibility in the target groups with similar characteristics as the subjects in the present study.

Although all 4 common tests showed excellent test-retest and interrater reliability, the emphasis of their measurements varied (Hansberger et al., 2019). The SLR and PKE were performed by prolonging the hamstring of the unilateral dominant lower limb whereas the SRT and TTT required maximum trunk flexion so that the measurement may be more influenced by the tightness of the posterior side of the trunk muscles and bilateral hamstrings (Hansberger et al., 2019). Furthermore, the proximal part of the hamstring was prolonged during the SLR test as it was performed on the flex hip joint with a straight leg. In contrast, the PKE measured the distal part of the hamstring by directly extending the knee joint with fixation of the hip. In addition, (Miyamoto et al., 2017), investigated the effects of hamstring stretching on passive muscle stiffness with straight leg raise and passive knee extension maneuvers using 12 male subjects. With ultrasound shear wave elastography, a passive knee extension stretching maneuver was shown to reduce the shear modulus in all parts of the hamstring involving the biceps femoris, semitendinosus (ST) and semimembranosus (SM) whereas the stretching effects of the passive straight leg raise maneuver were significant only in the ST and SM. This to a certain extent supported that the different parts of the hamstring were lengthened during the SLR and PKE tests. Therefore, these 4 tests might be used for different purposes or different statuses to measure hamstring flexibility.

It is worth noting that to measure hamstring flexibility using these 4 common tests, some strategies should be adopted to avoid compensation. For the SLR and PKE, lumbar lordosis may decrease as the lengthening of the hamstring may induce posterior tilting of the pelvis. This may influence the reliability and stability of the measurement results. Ayala and colleagues (2011) reported a higher validity of the SLR than previous studies that did not use a low-back protection support to minimize posterior pelvic tilt. Thus, a control strategy seeking to maintain the normal lordotic curve should be used (2015). In the current study, we placed a folded towel to prevent compensation for pelvic tilt during the SLR and PKE. Furthermore, to prevent pelvis tilting, it was necessary to stabilize the nondominant leg on the couch. In addition, the hip angle of the dominant leg throughout the PKE test should be blocked to 90 degrees with an obstacle to hold the thigh perpendicular to the couch for constant measurements. While conducting the SRT and TTT, the key point was to require the subjects to keep their knee in the full extension position throughout the test (Ayala et al., 2012). This needs to be emphasized to the participants before and during the tests.

A great number of previous studies have reported that the strength of thigh muscles plays a special role in hamstring injury. A meta-analysis demonstrated that high quadriceps muscle strength measured with isokinetic testing at 60°/s was a risk factor for hamstring injury with an effect size of 0.05–0.81 (Freckleton and Pizzari, 2013). (van Beijsterveldt et al., 2013) conducted a systematic review and showed that the muscle imbalance of isokinetic assessments of the hamstring and quadriceps significantly increased the rate of hamstring injury in soccer players. Although the isokinetic testing for measuring thigh muscle imbalance is an effective method of identifying at-risk individuals, due to several practical reasons forementioned it have the disadvantage of having a limited use in clinical practice. If a simple hamstring flexibility test that can be found to correlate with isokinetic performance imbalance of thigh muscles, it seems to be a convenient way to indicate the potential risk for hamstring injury when the isokinetic testing is not available. However, no relevant studies have been reported. Thus, the current study analyzed the relationship between the hamstring length measured with 4 flexibility tests and the isokinetic performance of the hamstring and quadriceps muscle and the H/Q ratio. The current results demonstrated the significant positive relationship for the SLR, SRT and TTT with the H/Q ratio of isokinetic performance in peak torque measured at 180°/s. In contrast, the PKE was also shown to be fairly correlated with the AP of the hamstring and all isokinetic parameters of the H/Q at an angular speed of 180°/s. Thus, this might suggest that the shortness of the hamstring as a possible risk factor for injury in college-age young adults might be more meaningful as indicated by the PKE test rather than other tests such as the SLR, SRT and TTT.

In the present study, it was noted that the significant correlations were found between the hamstring flexibility and the H/Q ration assessed only at higher speed with 180°/s but not at lower speed of 60°/s. Indeed, the most frequently reported strength imbalance ratio is the concentric H/Q ratio at an angular velocity of 60°/s (Mau-Moeller et al., 2019). However, previous studies have been inconclusive with respect to the optimal speed for testing the H/Q ratio as the risk factor for hamstring injury, with Orchard (1997) and Lee (2018) suggesting slower speed of 60°/s and others suggesting speeds of 180°/s or faster (Yeung et al., 2009; Lee et al., 2018). These studies involved participants at different levels of expertise and professionalism, which make comparison of the findings difficult. Hence, for the sample group of college-age students in this study, it remains to be verified if the predicating effect of H/Q ratio assessed at angular velocity of 180°/s on the risk of hamstring injury is more supperior than that at 60°/s.

Some limitations might restrict the interpretation of the current study. First, a total of 43 subjects were recruited to investigate the reliability of 4 common hamstring flexibility tests and their relationships with the isokinetic performance of thigh muscles. Thus, the effect sizes regarding the statistical analysis, which was significant, were relatively small based on this finite sample. Furthermore, many previous studies related to hamstring flexibility tests refer to athletes, some of them were also cited to support the view in this article. Whereas, all participants recruited in this study were healthy young adults, even though all of them play sports regularly. Hence, unidentical characteristics of samples may also limit the generalization of the results of current study. Therefore, whether a similar reliability of the hamstring flexibility demonstrated in the present study would be present when the 4 hamstring flexibility tests are applied to elite athletes in different disciplines or other patients with musculoskeletal disorders would require further investigation.

To conclude, this study confirmed that the SLR, PKE, SRT and TTT were reliable flexibility tests for hamstring muscles; and the PKE might be a more valid outcome measure to predict hamstring injury in healthy young adults. As the parameters of response stability for these 4 common tests, the SEM and MDD_95_ would contribute to interpreting the changes in hamstring flexibility in young adults in clinical practice.

## Data Availability

The original contributions presented in the study are included in the article/Supplementary Material, further inquiries can be directed to the corresponding authors.
